# Impact Load Localization Based on Multi-Scale Feature Fusion Convolutional Neural Network

**DOI:** 10.3390/s24186060

**Published:** 2024-09-19

**Authors:** Shiji Wu, Xiufeng Huang, Rongwu Xu, Wenjing Yu, Guo Cheng

**Affiliations:** 1 Laboratory of Vibration and Noise, Naval University of Engineering, Wuhan 430033, China; wsl202304@163.com (S.W.);; 2 National Key Laboratory of Vibration and Noise on Ship, Naval University of Engineering, Wuhan 430033, China

**Keywords:** shock source localization, convolutional neural network, multi-scale, impact loads

## Abstract

In order to achieve impact load localization of complex structures such as ships, this paper proposes a multi-scale feature fusion convolutional neural network (MSFF-CNN) method for impact load localization. An end-to-end machine learning model is used, where the raw vibration signals of impact loads are directly fed into the network model to avoid the process of feature extraction. Automatic feature learning and feature concatenation of the signal are achieved through four independent convolutional layers, each using a different size of convolutional kernel. Data normalization and L2 regularization techniques are introduced to enhance the data and prevent overfitting. Classification and localization of impact loads are accomplished using a softmax classification layer. Validation experiments are carried out using a ship’s stern compartment model. Our results show that the classification and localization accuracy of the impact load sample group of MSFF-CNN reaches 94.29% compared with a traditional CNN. The method further improves the ability of the network to extract state features, takes local perception and global vision into account, effectively improves the classification ability of the model, and has good prospects for engineering applications.

## 1. Introduction

In recent years, technological advancements have led to the widespread adoption of Structural Health Monitoring (SHM) systems in large-scale mechanical equipment and platforms. These systems are particularly adept at detecting and precisely localizing structural anomalies, which are often precipitated by impact events—key contributors to mechanical structural issues. In the maritime industry, the inevitability of sudden faults, such as the unexpected loosening of ship components, during navigation underscores the importance of prompt and accurate identification of the origins of these impact loads. Timely fault rectification is paramount for extending the operational lifespan of vessels. The integration of SHM systems, therefore, not only enhances the safety and reliability of maritime operations but also contributes significantly to the preservation and optimization of ship infrastructure.

In the quest for effective localization methods, the installation of sensors on ship structures has emerged as a viable approach. This involves the collection of signals generated by impact loads, followed by in-depth analysis and processing of these signals. The prevalent impact source localization techniques primarily focus on signal processing analysis and can be categorized into five main types. The first is time-difference-based localization [1,2,3,4]. This method utilizes time difference in the propagation of stress waves from an impact signal to various accelerometers. By calculating the focal length from sensor locations using the time difference, two sets of hyperbolic equations are derived, with their intersection point indicating the impact location [1]. This technique boasts advantages such as a low computational load and rapid processing speed. However, it is susceptible to inaccuracies in time difference extraction, including signal interference, channel crosstalk, weak and ambiguous arrivals, propagation delays, sensor location errors, high background noise, and simple hardware faults [5,6,7,8,9,10], which can lead to underestimated localization results. The second is optimization- and inversion-based localization [11,12,13,14]. Proposed by Xu, L., this method employs a transfer function based on eigenvectors for impact monitoring and localization [14]. It constructs a transfer function that relates sensor signals to the impact load’s time course to identify the location of the impact. The advantage of this method is that it does not require an accurate structural model. However, changes in structure can alter the transfer function of the structural response, and solving for ill-conditioned matrices demands significant computational resources, making real-time localization challenging. The third is acoustic holography and beamforming localization [15,16,17,18]. Xiao, D., utilized a beamforming method for acoustic source localization, employing two sensor arrays that were installed along two perpendicular directions to determine the *x* and *y* coordinates of the sound source [15]. This type of localization method offers high resolution and a capability to locate both silent and moving sound sources. However, it requires a certain number of sensors and specific arrangements, making it difficult to implement on a large scale. The fourth is circle intersection localization. Fang, L., introduced a circle intersection localization method that does not rely on time difference calculations to determine the distance from sensors to the localization point [19]. Instead, it uses the absolute distance from the sensors to the localization point for positioning. This method sidesteps challenges of accurately measuring the time difference in stress wave arrivals at various sensors but necessitates prior knowledge of the wave propagation speed, which can be difficult to obtain accurately in structures like cylinders, where vibration waves include transverse, longitudinal, and bending waves. The fifth is high-resolution spectral estimation localization. Schmidt employed a method that decomposes a signal into its characteristic space, separating it from the noise space, and uses their orthogonality to estimate the signal’s wave arrival direction [20]. This method offers high resolution and accuracy but involves substantial computational demands and performs poorly under low signal-to-noise ratios, making it less commonly used. Each of these methods has its unique strengths and limitations. The choice of technique often depends on the specific requirements and constraints of the application at hand.

The aforementioned localization methods are all based on accurately grasping the complex mapping relationship between the sensor signal and sound source. However, for a large ship structure, there is nonlinear mapping, and the structure is complex and large in scale, necessitating a clear understanding of the physical mechanisms behind these methods. In recent years, the convolutional neural network (CNN) has been introduced into sound source localization due to its powerful nonlinear modeling capabilities [21]. Toni, H., preprocessed data, using spectrograms as sample inputs to construct a CNN model, with the spatial direction and content type as the classification output categories [22]. Chakrabarty, S., designed a CNN to predict the azimuth of one or two speakers in a reverberant environment [23]. The input features were multi-channel short-time Fourier transform (STFT) phase spectrograms. Thuillier, E., studied the use of a CNN with binaural input features (same-side and opposite-side head-related transfer function magnitude responses) to estimate the elevation angle in isolation [24]. Fahim, A., applied an eight-layer CNN to first-order binaural input features for localizing multiple sources in a reverberant environment [25]. Vargas, E., used phase diagrams as sample inputs to construct a CNN model, with every 5° of the sound source arrival direction as an output category [26]. The aforementioned methods adopt a two-stage model of feature extraction plus classification localization, which has two issues. First, for sensors deployed on large structures such as ships, there is no phase information or binaural information, which makes it impossible to use phase diagrams or binaural characteristics as features. The impact source signals targeted in this paper do not have obvious spectral characteristics, making spectral features less effective as inputs. Second, feature extraction can lose some useful information from the original data, leading to difficulties in complex sound source identification and poor robustness of the localization model, which cannot meet the requirements for impact source localization on large structures such as ships.

A multi-scale feature fusion convolutional neural network (MSFF-CNN)-based method for localizing impact loads on complex structures, such as ship compartments, is proposed. Initially, the ship is simplified to a combined frustum–cylinder body, and a grid for the localized partitioning of impact loads on the ship compartment is constructed, transforming the localization issue into a classification problem. There is no need for pre-extracted features from the input data; an end-to-end approach [27,28] is adopted, where the original vibration signals of the impact load are directly input into the MSFF-CNN. Each scale operates independently, with the first convolutional layer having kernels of different sizes, namely 2 × 1, 16 × 1, 64 × 1, and 128 × 1, to account for both local perception and global vision. After multiple epochs of convolution and pooling, features extracted at various scales are fused through a feature concatenation layer, followed by a classification layer that outputs the category, with different categories representing the numbered localization grid areas (i.e., the localization regions). This completes the classification localization of the impact load and calculates the accuracy at both the sample and sample group levels. Localization experiments are conducted using a simplified stern model—a combined frustum–cylinder body—and comparisons are made with methods such as CNNs and three-scale CNNs, validating the effectiveness of the proposed method in this paper.

## 2. MSFF-CNN Method

### 2.1. CNN Theory

Typical convolutional neural networks (CNNs) primarily consist of a convolutional layer, activation layer, pooling layer, fully connected layer, and softmax layer. The convolutional layer, pooling layer, and activation layer are designed to map original data into a hidden feature space for feature extraction. Fully connected layers are tasked with mapping learned ‘distributed features’ to the sample label space. The softmax layer then determines the probability distribution of samples across different categories, facilitating the classification process. Additionally, the MSFF-CNN constructed in this paper performs feature extraction independently at each scale and subsequently merges these features through a concatenation layer.

(1) Convolutional layer

The role of the convolutional layer is to extract features from input data. Different convolutional kernels act as distinct feature extractors. The matrix values of the input receptive field are multiplied element-wise with the matrix values of the convolutional kernel, and after summing these products and adding a bias, the result is passed through an activation function to obtain the feature map. The mathematical model is represented as follows:

(1) $$s_{i,j}=x_{i,j}w_{i,j}=f\left(\sum_{m}\sum_{n}x_{i+m,j+n}w_{m,n}+w_{b}\right)$$

In this equation, *s_i,j_* represents the element at the *i* row and *j* column of the feature map. *x_i,j_* represents the element at the *i* row and *j* column of the output matrix. *w_m,n_* represents the weight of the *m* row and *n* column of the convolutional kernel, and *w_b_* represents the bias term of the convolutional kernel. *f* denotes the activation function (this paper selects the Relu function as its activation function). The Relu function is defined as *f*(*x*) = max (0, *x*), and its derivative is as follows:

(2) $$f^{\prime }(x)=\{\begin{array}{ll} 0,\;x<0 & \\ 1,\;x\geq 0 & \end{array}$$

(2) Pooled horizon

The pooling layer, commonly referred to as the subsampling or downsampling layer, is a typical operation in a CNN. It is often used after the convolutional layer to reduce the dimensionality of features output by convolutional layer. This not only effectively reduces the number of network parameters but also helps prevent overfitting. Taking the max pooling as an example, the formula is as follows:

(3) $$y_{i,j}=\max(x_{2i-1,2j-1},\;x_{2i-1,2j},\;x_{2i,2j-1},\;x_{2i,2j})$$

In this formula, *y_i,j_* represents the element at the *i*-th row and *j*-th column of the output matrix. *x*_2*i*−1,2*j*−1_, *x*_2*i*−1,2*j*_, *x*_2*i*,2*j*−1_, and *x*_2*i*,2*j*_ denote elements of the input feature map corresponding to their respective rows and columns.

(3) Fully connected layer

The fully connected layer is where every node is connected to all nodes in the preceding layer, serving to integrate features that were extracted earlier. The feature map (matrix) obtained from the last convolutional layer is flattened into a one-dimensional vector, providing input for the classifier.

(4) Softmax classification

The softmax function takes an *N*-dimensional vector of random true values as input and outputs another *N*-dimensional vector of true values, with values ranging in the (0, 1) range and summing up to 1.0. Through the softmax layer, the probability distribution of the current sample belonging to different classes can be obtained, which is a mapping process.

(4) $$S(a):\left[\begin{array}{c} a_{1}\\ a_{2}\\ \ldots \\ a_{N} \end{array}\right]\to \left[\begin{array}{c} S_{1}\\ S_{2}\\ \ldots \\ S_{N} \end{array}\right]$$

The formula for each element is as follows:

(5) $$S_{j}=\frac{e^{a_{j}}}{\sum_{k=1}^{N}e^{a_{k}}}\forall j\in 1\ldots N$$

(5) Concat feature series layer

The role of the concat layer is to concatenate two or more feature maps along a certain dimension, thereby creating a larger feature map. This concatenation typically occurs along the scale dimension and does not involve element-wise operations.

### 2.2. MSFF-CNN Model

**Figure 1:**
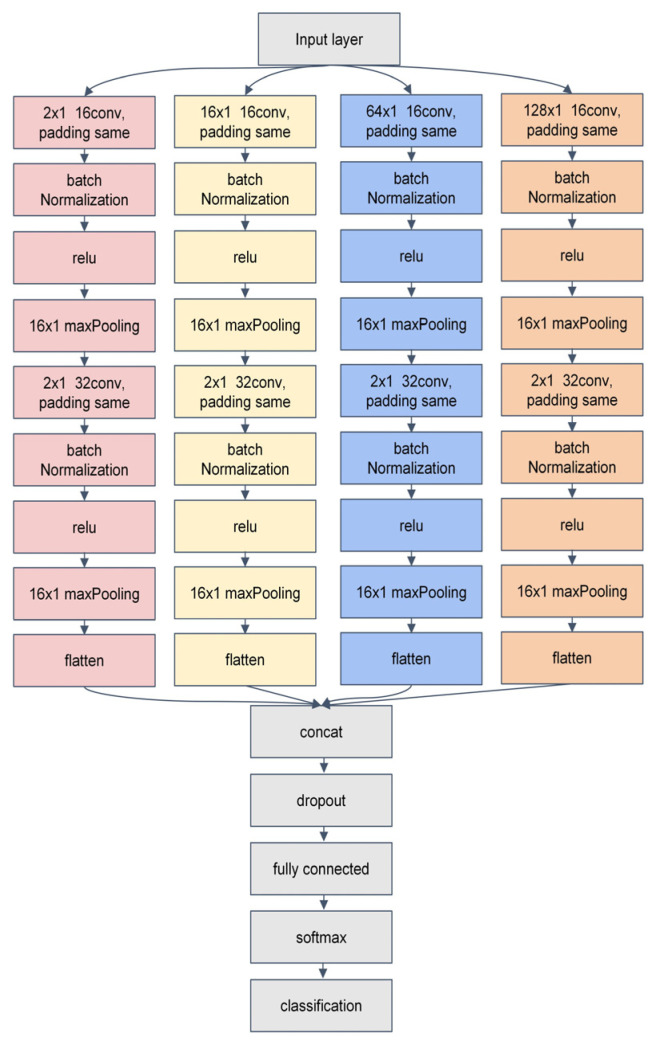
Multi-scale convolutional neural network structure.

To fully leverage the advantages of a CNN in signal feature extraction and enhance the model’s anti-interference capability, this paper proposes the MSFF-CNN method for impact load localization based on a CNN. Each scale consists of two sets of convolutional layers, batch normalization layers, Relu activation layers, and max pooling layers, followed by a flattening operation. Features extracted from the four scales are merged into the concatenation layer, and then, the model goes through dropout, fully connected layers, and finally softmax classification to output results. The MSFF-CNN model’s structure is shown in Figure 1, and compared to traditional CNNs, the main improvements are described in the following paragraph.

Transitioning from single-scale optimization of a traditional CNN to a multi-scale approach, where each scale operates independently before feature concatenation, performing automatic feature extraction separately, enhances the feature extraction capability. The first convolutional layer of four scales has convolutional kernel sizes that vary, specifically 2 × 1, 16 × 1, 64 × 1, and 128 × 1. Smaller kernel sizes can extract local features, while larger kernel sizes can expand the receptive field, balancing local perception with a global perspective. The pooling layer has a size of 16 × 1, which is moderate and effective in preserving valuable information during the feature extraction process. Two sets of convolutional layers have different convolutional depths. The first layer has a depth of 16, and the second layer has a depth of 32, yielding different feature mappings for a set of input data, balancing the model’s parameters, complexity, and feature extraction capability. The introduction of data standardization and L2 regularization, along with the shuffling of datasets during training, is employed. Data standardization is introduced for data enhancement, L2 regularization is used to suppress overfitting phenomena, and the shuffling of dataset prevents under-training of certain localization areas.

### 2.3. Impact Load Location Method Based on MSFF-CNN

**Figure 2:**
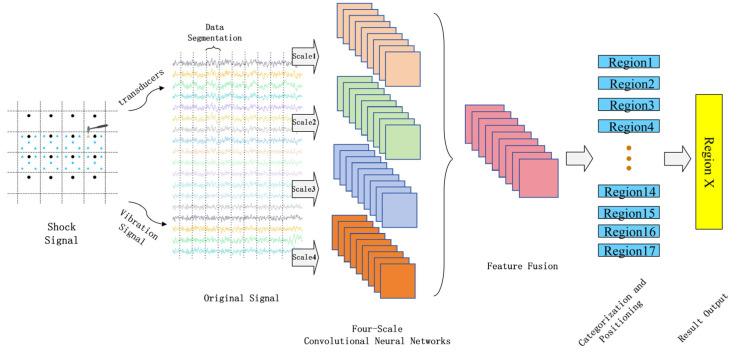
Multi-scale convolutional neural network localization flowchart.

An abnormal impact signal collected by the sensor in its original time-domain form is directly input into the MSFF-CNN. The raw signal passes through the convolutional and pooling layers, achieving automatic feature extraction of the original signal. Subsequently, four sets of obtained features are fused, and the softmax classifier is used for classification to complete the localization of the impact load area. The steps for localization based on the MSFF-CNN are as follows: establishment of the localization model, dataset collection and construction, construction and training of the MSFF-CNN, and classification and localization of the impact load, as shown in Figure 2.

Firstly, an impact load test model was constructed for the ship’s stern test platform, where the model was meshed and the grids were numbered, thereby transforming the positioning problem into a classification problem. Subsequently, sensors were utilized to collect the original impact signals within the positioning model, and the raw data were labeled in conjunction with the model from step one, serving as input for the MSFF-CNN. Secondly, the MSFF-CNN model was established, with the weights and biases of the MSFF-CNN model being randomly initialized. Training parameters, such as the initial learning rate, were set, and the dataset was shuffled (with a training set to test the set ratio of 7:3). The network parameters were adjusted based on the output results until the training criteria were met. Finally, through the aforementioned steps of model construction, data collection, and network training, the network outputs category identifiers, with each identifier corresponding to a specific area, thus completing the classification and localization of the impact load.

## 3. Experimental Verification and Analysis

### 3.1. Experimental Settings

**Figure 3:**
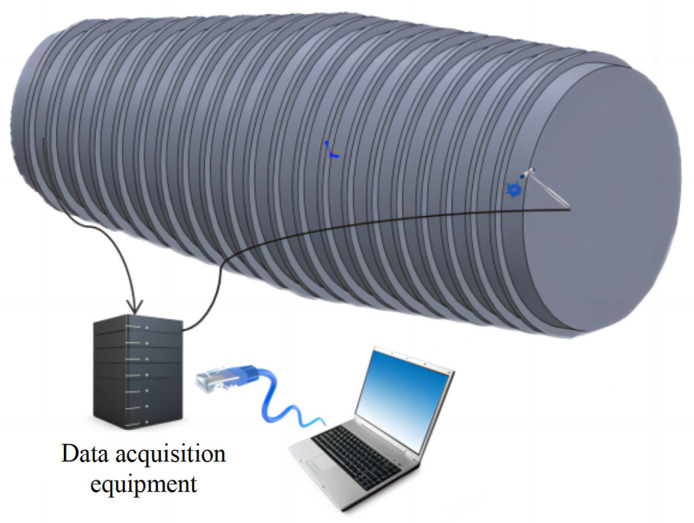
Schematic diagram of knockout site during model test.

**Figure 4:**
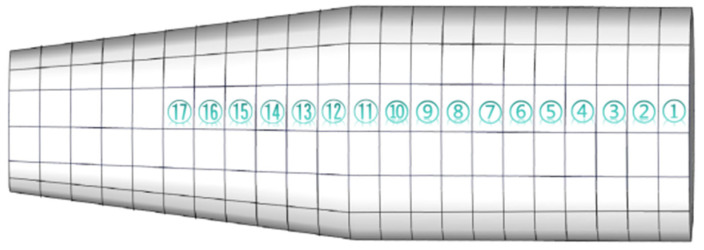
Schematic diagram of modeling mesh partitioning.

**Figure 5:**
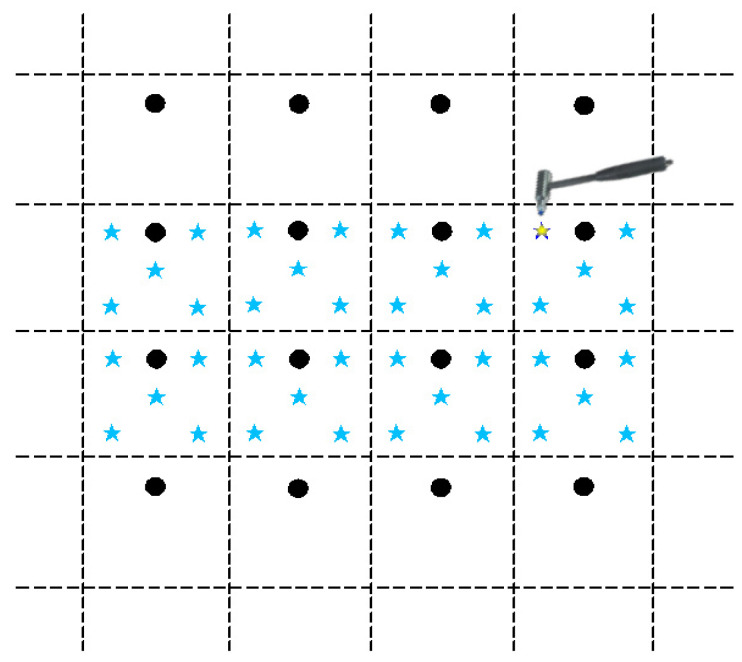
Schematic diagram of sensor arrangement and location of knocking point (The star represents the tapping position and the dot represents the sensor arrangement position).

**Figure 6:**
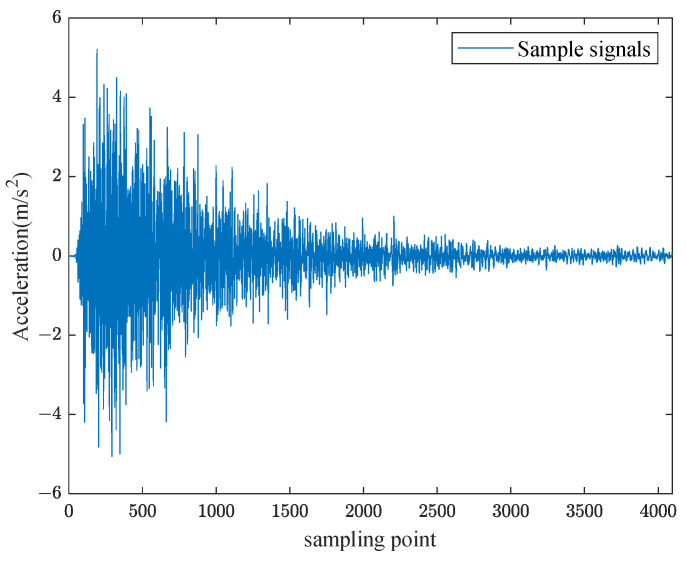
Raw acquisition data entry division.

**Figure 7:**
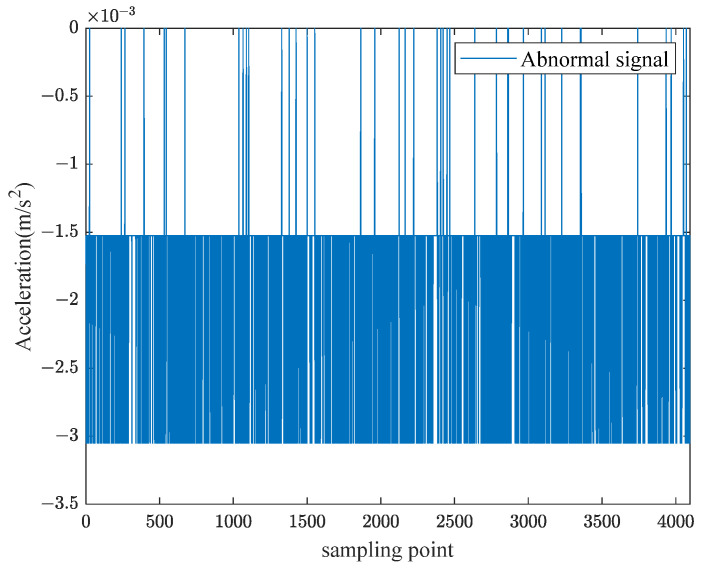
Abnormal sample signal.

The experimental setup includes one rigid force hammer, two data acquisition devices, one laptop computer, and ninety-four vibration acceleration sensors. The test schematic is shown in Figure 3. Based on the test model of a ship’s stern, an impact load classification and localization model is established. The test model of a ship’s stern is simplified to a combination of a cylinder and cone, with the grid divided horizontally each 0.8 m and vertically each 1 m. The surface of the model is partitioned, and grid areas are numbered, as shown in Figure 4. A rigid force hammer is used to strike the surface of the model structure to simulate the impact of abnormal loads. In Figure 4, each of the squares numbered from ① to ⑰ is struck at five different positions, as indicated by the blue asterisks in Figure 5. Each point is struck three times, resulting in a total of 255 sets of data. In Figure 5, a vibration acceleration sensor is placed in each area, as shown by black circles. Each strike is accompanied by 1 s data collection with a sampling frequency of 13,183 Hz, and the obtained sensor signal data represent the vibration acceleration. A total of 4096 sampling points before and after the occurrence of an impact load, approximately 0.31 s of data, are taken as input samples, as shown in Figure 6. Each sensor serves as an independent sample, and each data sample is labeled, with each label corresponding to the area where the impact load occurred. The total number of samples is 23,970 (255 sets × 94 sensors), and samples with anomalies are excluded, as shown in Figure 7. Ultimately, 21,714 samples are selected for training and validation, with the training and testing sets having a ratio of 7:3.

### 3.2. MSFF-CNN Parameter Settings

**Figure 8:**
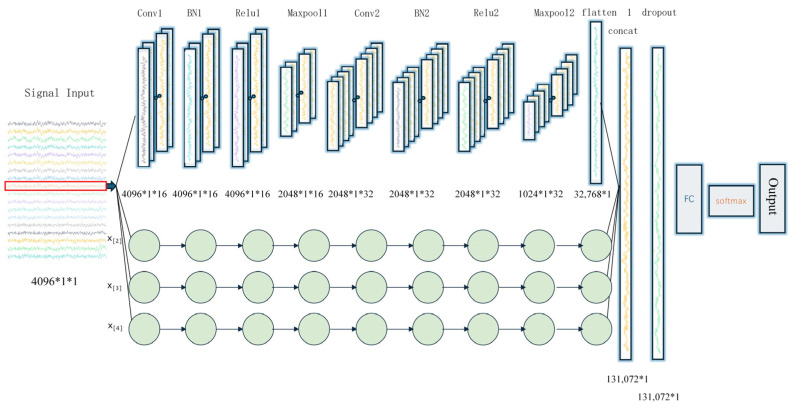
Internal structure of multi-scale convolutional neural network.

In convolutional neural networks, the parameter settings have a significant impact on the accuracy of results. For the multi-class classification task in this paper, parameters affecting the model’s performance, such as the size of convolutional kernels, *S*, the depth, *F*, and the size of the pooling layers, *K*, were selected. Through repeated experiments, the final model parameters were determined, as shown in Table 1. The internal structure of the MSFF-CNN is illustrated in Figure 8.

### 3.3. Positioning Result

The original dataset is input into the MSFF-CNN constructed in this paper, where the data undergo a series of operations, including automatic standardization, convolution, pooling, and L2 regularization, to automatically extract features. In this paper, the input data are standardized using the Z-score method, which transforms data into a standard normal distribution with a mean of 0 and variance of 1. The formula is as follows:

(6) $$N=\frac{D-mean}{std}$$

In this context, *N* represents the data after Z-score standardization, *D* represents the data before standardization, *mean* represents the mean of the data before standardization, and *std* represents the standard deviation of the data before standardization. The purpose of this treatment is to identify and correct errors, ambiguities, and missing issues in the data and transform complex and non-standard data into concise and standardized data, thereby improving the quality and operability of the data.

The adoption of an L2 regularization strategy is chosen to prevent overfitting and enhance the model’s generalization capability. The core idea of L2 regularization is to restrict the range of model parameter values. Parameters with a large range of values may only be catering to the training set, and they can amplify noise in samples that are input to the model, leading to distorted output results.

This paper uses accuracy as the validation metric for the network model. For multi-class classification, accuracy represents the percentage of samples in a test set that are correctly classified out of total number of samples, which is an important metric for measuring a model’s performance. The formula is as follows:

(7) $$A=\frac{C}{I}$$

In this context, *A* represents accuracy, *C* represents the number of correctly classified samples, and *I* represents the total number of input samples.

In engineering applications, a group of 94 sensor samples constitutes a sample group corresponding to a single category. When outputting, the category that appears most frequently within a sample group is taken as the output of the sample group. The accuracy is then defined as the percentage of correctly classified sample groups in the test set out of the total number of sample groups. The formula is as follows:

(8) $$B=\frac{E}{M}$$

In this context, *B* represents accuracy, *E* represents the number of correctly classified sample groups, and *M* represents the total number of input sample groups.

**Figure 9:**
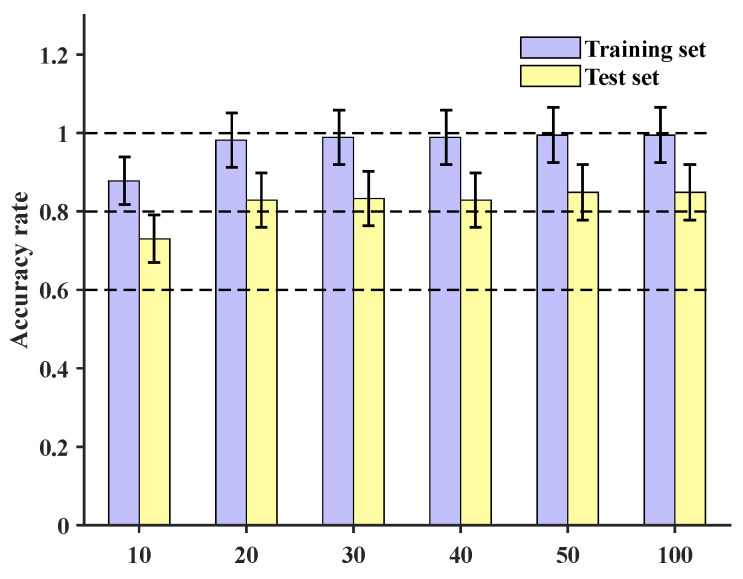
The accuracy of the training set and test set of the MSFF-CNN constructed in this paper under different numbers of training epochs.

Training was conducted for different numbers of epochs, specifically 10, 20, 30, 40, 50, and 100. At the sample level, the final accuracies of the training set and test set are depicted in Figure 9. These results indicate that the localization accuracy reached 84.9%.

**Figure 10:**
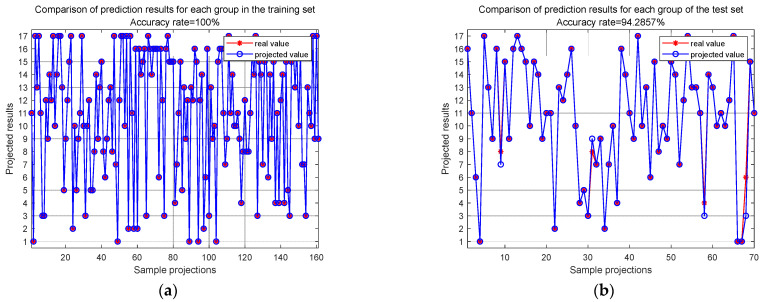
MSFF-CNN’s accuracy for training set group and test set group at 100 epochs: (**a**) training set group accuracy at 100 epochs; (**b**) test set accuracy at 100 epochs.

At the sample group level, after 100 epochs of training, the final accuracies of the training set and test set are depicted in Figure 10. These results show that the training set localization accuracy is 100%, and the test set localization accuracy reaches 94.29%. This indicates that the MSFF-CNN constructed in this paper has significant potential for engineering applications.

Without the adoption of standardization, after 100 epochs of training, at the sample level, the training set localization accuracy was 99.35%, and the test set localization accuracy reached 78.75%, which is lower than the 84.89% achieved with standardization included. At the sample group level, the training set localization accuracy was 100%, and the test set localization accuracy reached 91.43%, which is lower than the 94.29% achieved with standardization included, indicating that standardizing input data has a certain data enhancement effect.

Without the application of L2 regularization, after 100 epochs of training, at the sample level, the training set localization accuracy was 99.58%, and the test set localization accuracy reached 78.69%, which is lower than the 84.89% achieved with L2 regularization included. At the sample group level, the training set localization accuracy was 100%, and the test set localization accuracy reached 90.00%, which is lower than the 94.29% achieved with L2 regularization included, indicating that applying L2 regularization during network training prevents overfitting to some extent.

**Figure 11:**
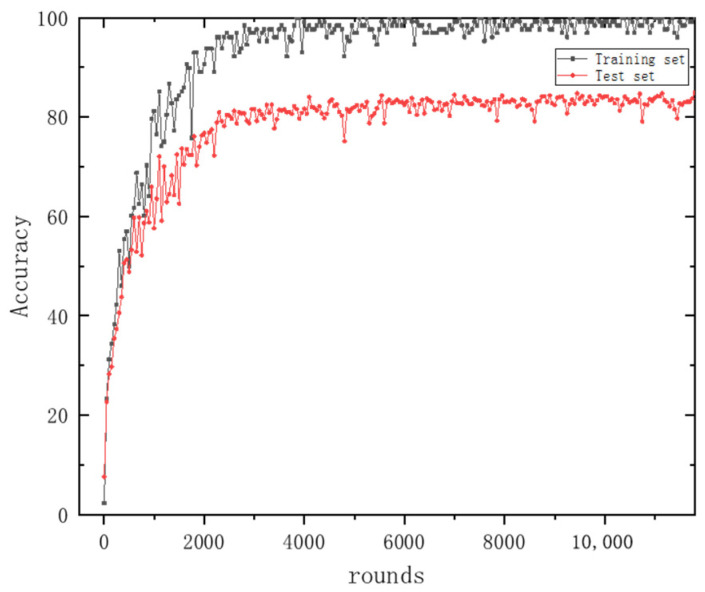
The accuracy of the MSFF-CNN training set and test set changes with the number of iterations.

**Figure 12:**
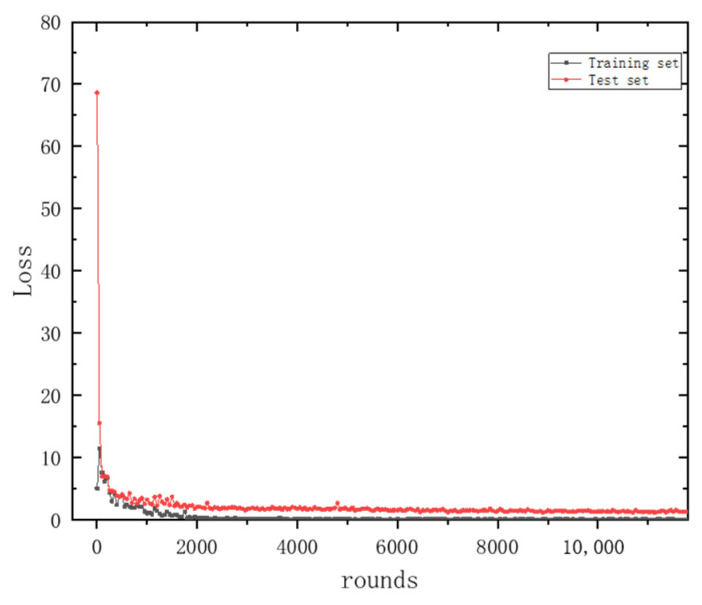
MSFF-CNN training set and test set loss function change trend with number of iterations.

Figure 11 and Figure 12 depict the variations in the accuracy and loss function for the training and testing datasets, respectively. Here, the horizontal axis ‘rounds’ refers to the number of iterations. In other parts of the text, ‘epochs’ denotes the number of times the entire training dataset is completely traversed (i.e., used for training) by the neural network, with one epoch typically encompassing multiple rounds. From these figures, it can be observed that the accuracy and loss function values for both the training and testing datasets tend to stabilize with an increase in the number of iterations (rounds), indicating that the method presented in this paper possesses strong stability.

### 3.4. Contrastive Analysis

**Figure 13:**
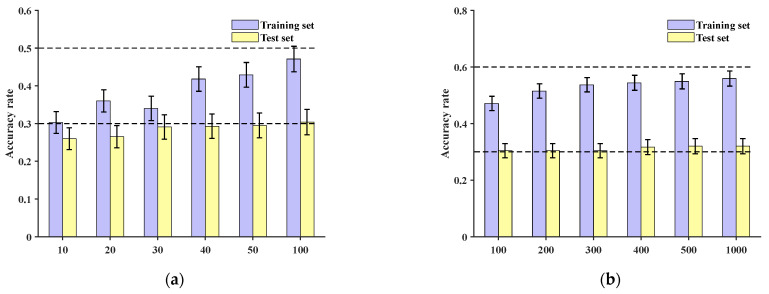
Training set and test set accuracy of conventional CNN with different numbers of training epochs: (**a**) accuracy of training set and test set under 100 epochs; (**b**) accuracy of training set and test set between 100 and 1000 epochs.

Comparing the method presented in this paper with the CNN model from reference [27], original data are directly inputted. At the sample level, training is conducted for 10, 20, 30, 40, 50, and 100 epochs, respectively. The accuracies of the training and test sets are shown in Figure 13a, with the test set accuracies all being below 30%. Continuing to increase the number of training epochs to 200, 300, 400, 500, and 1000, the test set accuracies remain around 30%, as depicted in Figure 13b.

**Figure 14:**
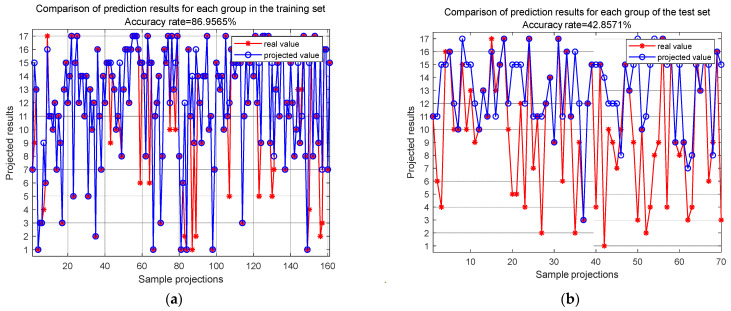
CNN accuracy of training set group and test set group at 100 epochs: (**a**) accuracy of training set at 100 epochs; (**b**) accuracy of test set at 100 epochs.

At the sample group level, after 100 epochs of training, the final accuracies of the training set and test set are depicted in Figure 14. These results demonstrate that the training set’s localization accuracy is 86.96%, while the test set’s localization accuracy is only 42.86%. This indicates that under the conditions described in this paper, the network is unable to effectively accomplish classification and localization of impact loads.

**Figure 15:**
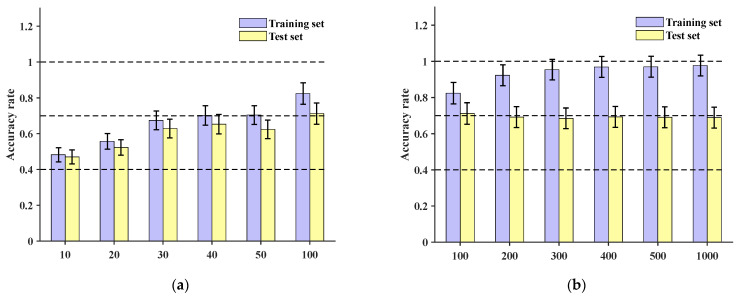
Training set and test set accuracy of three-channel MCCNN with different numbers of training epochs: (**a**) accuracy of training set and test set under 100 epochs; (**b**) accuracy of training set and test set between 100 and 1000 epochs.

Comparing the method presented in this paper with the MC-CNN model (multi-channel convolutional neural network), the original dataset was input into the MC-CNN [29]. The data underwent a series of operations such as convolution and pooling to automatically extract features. Training was conducted for 10, 20, 30, 40, 50, and 100 epochs, respectively, and the final accuracies for the training set and test set are shown in Figure 15a, with the maximum accuracy reaching 71.2%. Further increasing the number of training epochs to 200, 300, 400, 500, and 1000, the test set accuracy remained below 70%, as depicted in Figure 15b.

**Figure 16:**
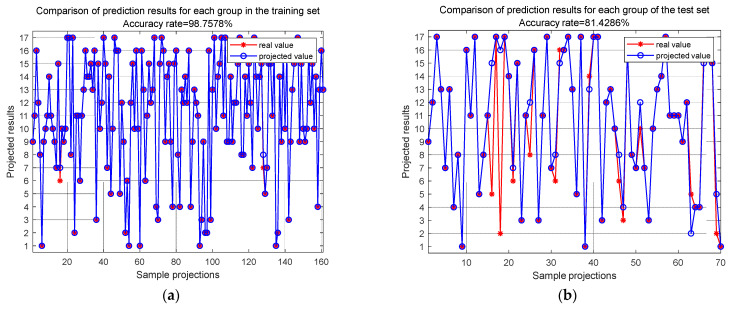
MCCNN accuracy for training set group and test set group at 100 epochs: (**a**) accuracy of training set at 100 epochs; (**b**) accuracy of test set at 100 epochs.

At the sample group level, after 100 epochs of training, the final accuracies of the training set and test set are shown in Figure 16. These results indicate that the training set’s localization accuracy is 98.76%, and the test set’s localization accuracy is 81.43%.

In summary, at the sample level, as illustrated in Figure 15 and Table 2, the CNN achieved a test set accuracy of 32.0% after 1000 training epochs. The MC-CNN reached a test set accuracy of 68.9% after 1000 training epochs, and the MSFF-CNN constructed in this paper achieved a test set accuracy of 84.9% after only 100 training epochs.

**Figure 17:**
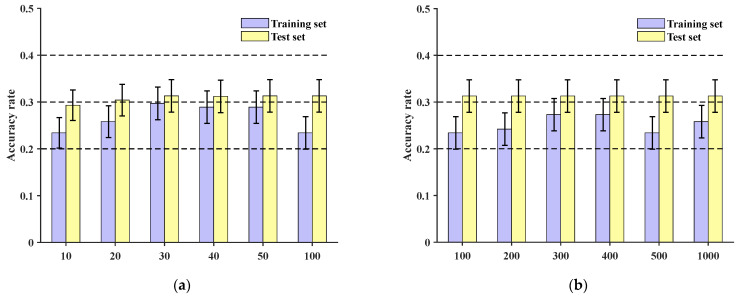

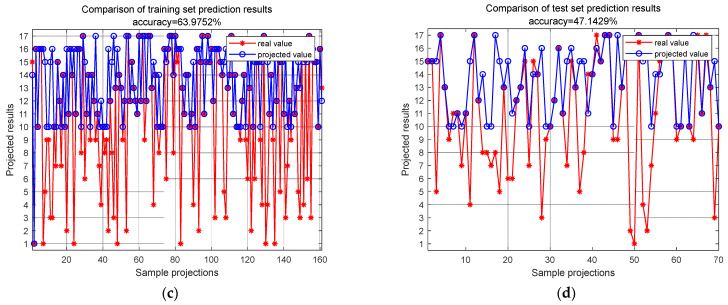
Accuracy of training set and test set under different epochs of MuCNN model: (**a**) training set and test set accuracy under 100 epochs; (**b**) training and test set accuracy between 100 and 1000 epochs; (**c**) training set accuracy at 100 epochs; (**d**) test set accuracy at 100 epochs.

The proposed method was compared with the multi-scale one-dimensional convolutional neural network model in reference [30], which was referred to as the MuCNN for short. The original dataset was input into the model, and the data underwent a series of operations such as convolution and pooling to complete automatic feature extraction. Also, after 10 epochs, 20 epochs, 30 epochs, 40 epochs, 50 epochs, 100 epochs, 200 epochs, 300 epochs, 400 epochs, 500 epochs, and 1000 epochs of training, the final maximum accuracies of the training set and the test set are 43.85% and 31.26%, respectively. At the sample group level, the accuracies of the final training set and test set were 63.98% and 47.14%, respectively, after 100 epochs of training, as shown in Figure 17.

**Figure 18:**
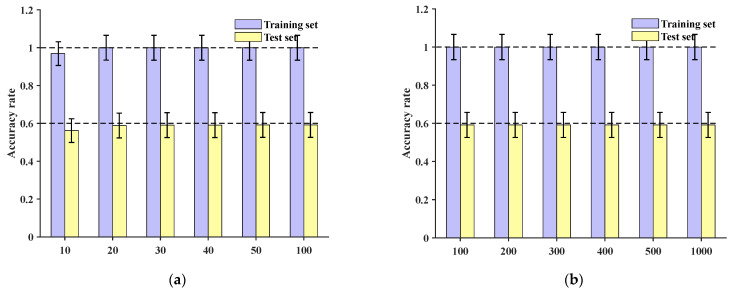

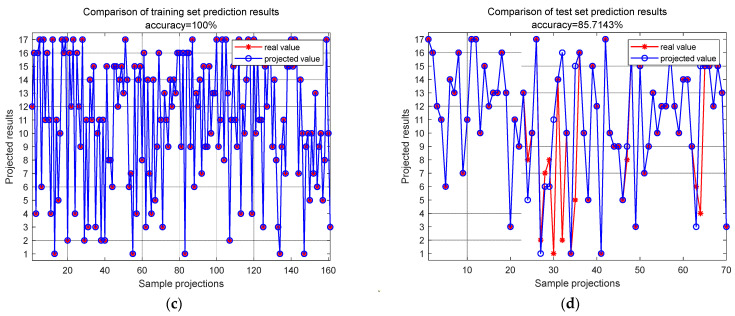
Accuracy of training set and test set under different epochs of MS-1DCNN model: (**a**) training set and test set accuracy under 100 epochs; (**b**) training and test set accuracy between 100 and 1000 epochs; (**c**) training set accuracy at 100 epochs; (**d**) test set accuracy at 100 epochs.

The proposed method is compared with the MS-1DCNN (multi-scale one-dimensional convolutional neural network model) presented in reference [31], and the original dataset is input into the model. After a series of operations such as convolution and pooling, automatic feature extraction is completed. Also, 10 epochs, 20 epochs, 30 epochs, 40 epochs, 50 epochs, 100 epochs, 200 epochs, 300 epochs, 400 epochs, 500 r epochs, and 1000 epochs of training are conducted. The final maximum accuracy rates of the training set and test set are 100% and 59.18%, respectively. At the sample group level, after 100 epochs of training, the accuracies of the final training set and test set were 100% and 85.71%, respectively, as shown in Figure 18.

**Figure 19:**
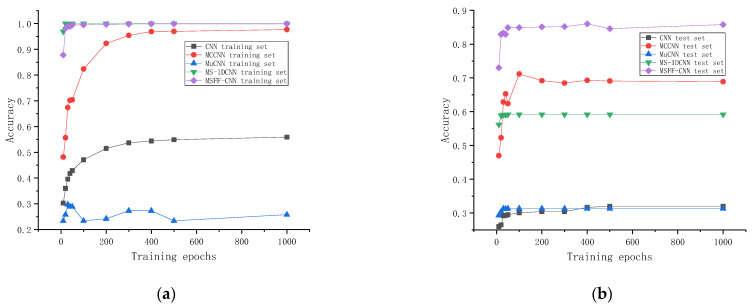
Comparison of the accuracies of the three methods with different numbers of training epochs and test epochs: (**a**) comparison of the accuracy of the three methods with different numbers of training epochs; (**b**) comparison of the accuracies of the three methods with different numbers of test epochs.

In summary, at the sample level, as illustrated in Figure 19 and Table 2, the CNN achieved a test set accuracy of 32.0% after 1000 training epochs. The MC-CNN reached a test set accuracy of 68.9% after 1000 training epochs, and the MSFF-CNN constructed in this paper achieved a test set accuracy of 84.9% after only 100 training epochs.

At the sample group level, as shown in Table 3, the CNN achieved a test set accuracy of 42.86% after 100 training epochs. The MC-CNN reached a test set accuracy of 81.43% after 100 training epochs. The MuCNN reached a test set accuracy of 47.14% after 100 training epochs. The MS-1DCNN reached a test set accuracy of 85.71% after 100 training epochs. The MSFF-CNN constructed in this paper achieved a test set accuracy of 94.29% after 100 training epochs. This indicates that the test set accuracy of the MSFF-CNN constructed in this paper is higher than the test set accuracies of both the CNN and MC-CNN.

**Figure 20:**
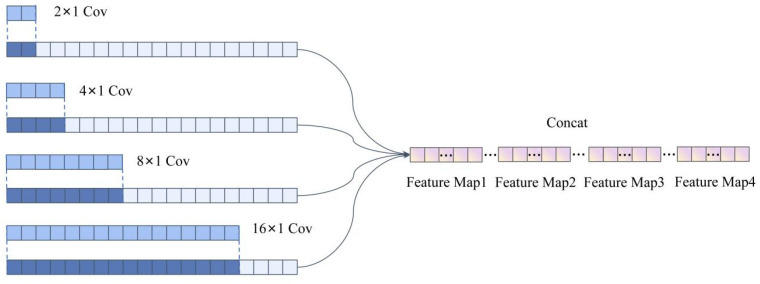
Multi-scale fusion convolutional neural network feature fusion diagram.

The accuracy of the multi-scale feature fusion convolutional neural network (MSFF-CNN) constructed in this paper exceeds that of the CNN and MCCNN owing to the multi-scale convolutional neural network’s feature concatenation across feature maps obtained at various scales, resulting in a larger feature map. This larger feature map encompasses the information from the feature maps derived at the original scales, where each scale’s feature map, due to the different settings of the convolutional kernel sizes, contains both local and global information. Small-sized convolutional kernels are capable of extracting local information, while larger kernels can capture extensive or even global information. The following figure illustrates the schematic of feature extraction and feature fusion. Concurrently, the depth of the convolutional layers and the size of the pooling layers have been optimized for selection, balancing the model’s parameters, complexity, and feature extraction capabilities. Additionally, data normalization and L2 regularization techniques were introduced to enhance the data and prevent the occurrence of overfitting phenomena, as shown in Figure 20.

### 3.5. Visual Analysis of Impact Load Classification and Positioning

**Figure 21:**
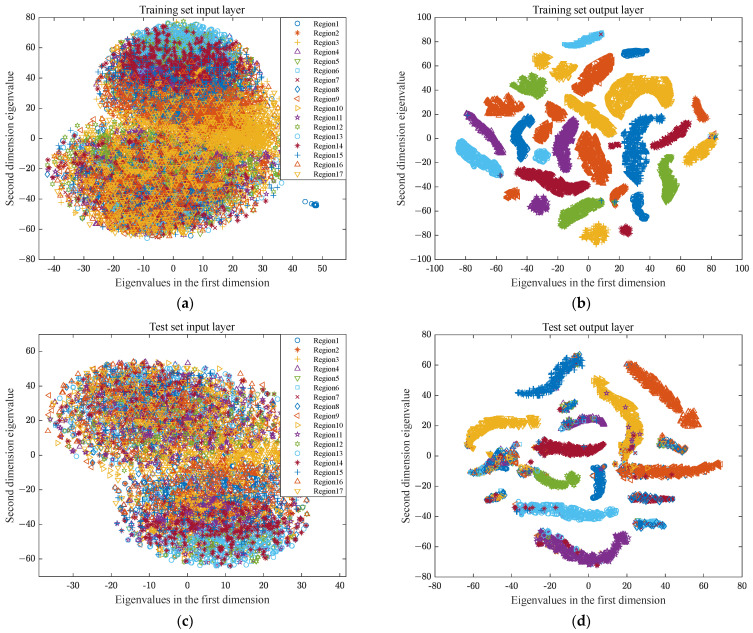
Visualization results for each layer of training set and test set: (**a**) visualization of training set’s input layer results; (**b**) visualization of training set’s output layer results; (**c**) visualization of test set’s input layer results; (**d**) visualization of test set’s output layer results.

The t-distribution stochastic neighbor embedding (t-SNE) algorithm is a nonlinear dimensionality reduction technique used for visualizing high-dimensional data [32]. To further analyze the effectiveness of the method presented in this paper, t-SNE was employed to perform secondary processing on features extracted from the dataset by the MSFF-CNN model to obtain more important and sensitive feature information. These obtained results are shown in Figure 21. Figure 21a–c represent the two-dimensional feature distribution status of the training set data extracted at the input layer, softmax layer, and output layer of the MSFF-CNN after t-SNE processing. It can be observed that original signals are interwoven at the input, with numerous samples mixed together, making it impossible to distinguish clear classification features, as shown in Figure 21a. The original data, after being input into the MSFF-CNN, undergo feature extraction layer-by-layer. As the depth increases, up to the softmax layer, data from the same category have already gathered together, and different categories are clearly clustered, as shown in Figure 21b. Figure 21c,d represent the two-dimensional feature distribution status of the test set data extracted at the input layer, softmax layer, and output layer of the MSFF-CNN after t-SNE processing. They exhibit similar distribution characteristics to the training set data, indicating that the clustering effects of both the training and test sets are significant, and also directly reflecting a clear classification effect.

## 4. Conclusions

Addressing the problem of impact load localization for complex structures such as ships, this paper proposes a localization method based on a multi-scale feature fusion convolutional neural network (MSFF-CNN). The MSFF-CNN model utilizes convolutional kernels of varying sizes at each scale, balancing local perception with a global perspective, and incorporates data standardization and L2 regularization, effectively enhancing the model’s classification capability. The localization experiments were conducted using a simplified stern model—a combination of a cylinder and cone. The experimental results indicate the following:

The MSFF-CNN method proposed in this paper has evolved from single-scale optimization to multi-scale optimization, enabling adaptive processing of the original vibration signals. Multiple scales independently perform feature extraction, enhancing the network model’s feature extraction capability, and after fusion, impact load classification localization is achieved.

After adopting data standardization, the localization accuracy rate was improved from 91.43% to 94.29%. When L2 regularization was applied during network training, the localization accuracy rate was improved from 90.00% to 94.29%, enhancing the model’s classification ability.

Compared with the CNN, MC-CNN, MuCNN, and MS-1DCNN, the constructed MSFF-CNN uses convolutional kernels of different sizes at each scale, further enhancing the network’s ability to extract state features and balancing local perception with a global perspective. The localization accuracy rate was improved from 42.86%, 81.43%, 47.14%, and 85.71% to 94.29%, demonstrating a promising prospect for engineering applications.

## Figures and Tables

**Table 1:** MSFF-CNN’s structural parameters.

Framework	Parameters			
	CNN1	CNN2	CNN3	CNN4
Conv1	*S* = 2 × 1 *F* = 16	*S* = 16 × 1 *F* = 16	*S* = 64 × 1 *F* = 16	*S* = 128 × 1 *F* = 16
BN1	Batch normalization1			
Relu1	Relu 1			
Maxp1	*K* = 16 × 1	*K* = 16 × 1	*K* = 16 × 1	*K* = 16 × 1
Conv2	*S* = 2 × 1 *F* = 32	*S* = 2 × 1 *F* = 32	*S* = 2 × 1 *F* = 32	*S* = 2 × 1 *F* = 32
BN2	Batch normalization 2			
Relu2	Relu 2			
Maxp2	*K* = 16 × 1	*K* = 16 × 1	*K* = 16 × 1	*K* = 16 × 1
Flatten	Flatten layer			
Concat	Concat layer			
Dropout	Probability: 0.5			
FC	Fully connected			
Softmax	Categorization			

**Table 2:** Accuracy of each method at the sample level.

Epochs	CNN		MCCNN		MuCNN		MS-1DCNN		MSFF-CNN	
	Training Set	Test Set	Training Set	Test Set	Training Set	Test Set	Training Set	Test Set	Training Set	Test Set
10	30.35%	26.02%	48.21%	47.03%	23.44%	29.33%	96.88%	56.16%	87.88%	73.05%
20	35.99%	26.52%	55.72%	52.33%	25.78%	30.36%	100%	58.91%	98.21%	82.89%
30	39.56%	29.17%	67.42%	62.94%	29.69%	31.25%	100%	59.10%	98.94%	83.33%
40	41.79%	29.25%	70.28%	65.31%	28.91%	31.23%	100%	59.12%	98.88%	82.90%
50	42.91%	29.45%	70.42%	62.43%	28.91%	31.26%	100%	59.18%	99.50%	84.89%
100	47.10%	30.36%	82.43%	71.21%	23.44%	31.26%	100%	59.18%	99.54%	84.89%
200	51.54%	30.41%	92.34%	69.22%	24.22%	31.26%	100%	59.18%	99.71%	85.10%
300	53.72%	30.37%	95.41%	68.51%	27.34%	31.26%	100%	59.18%	99.78%	85.24%
400	54.37%	31.75%	96.91%	69.33%	27.34%	31.26%	100%	59.18%	99.92%	85.96%
500	54.94%	32.02%	97.03%	69.12%	23.44%	31.26%	100%	59.18%	99.88%	84.57%
1000	55.87%	32.04%	97.72%	68.94%	25.78%	31.26%	100%	59.18%	99.89%	85.82%

**Table 3:** Accuracy of each method at the sample group level.

Serial Number	Methodology	Epochs	Accuracy of Training Set	Accuracy of Test Sets
1	CNN	100	86.96%	42.86%
2	MCCNN	100	98.76%	81.43%
3	MuCNN	100	63.89%	47.14%
4	MS-1DCNN	100	100.00%	85.71%
5	MSFF-CNN	100	100.00%	94.29%

## Data Availability

The original contributions presented in this study are included in the article material, and further inquiries can be directed to the corresponding author.
